# Shortening of ventriculoatrial interval after ablation of an accessory pathway

**Published:** 2006-10-01

**Authors:** Mohammad Alasti, Abolfath Alizadeh, Majid Haghjoo, Zahra Emkanjoo, Mohammad Ali Sadr-Ameli

**Affiliations:** Department of Pacemaker and Electrophysiology, Rajaie Cardiovascular Medical Center, Vali-asr Avenue, Tehran, IRAN

**Keywords:** ablation, short PR-long RP tachycardia, slowly conducting accessory pathway

## Case Presentation

**Figure 1:**
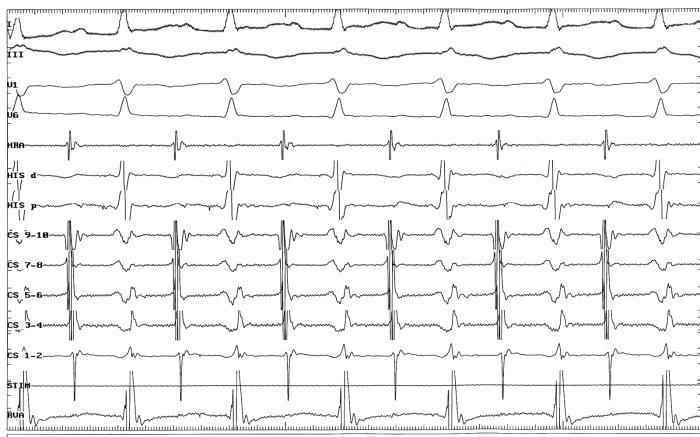
Surface leads and intracardiac electrograms during the arrhythmia are shown. HRA: high right atrium, CS: coronary sinus, His d: His distal, His p: His proximal, RVA: right ventricle apex. Recording speed: 100 mm/sec.

A 21-year old man with history of 8 year palpitation was referred for electrophysiologic study and possible radiofrequency ablation. Physical examination and transthoracic echocardiographic study did not disclose any abnormality. Baseline ECG showed normal sinus rhythm with normal PR and QRS intervals and no evidence of preexcitation. Antiarrhythmic drugs (propranolol and verapamil) were discontinued two days before the procedure. Baseline intervals in sinus rhythm were as follows: sinus cycle length=690 msec, AH=74 msec, HV=37 msec, QRS=90 msec, PR=133 msec. The minimal pacing cycle length maintaining 1:1 antegrade conduction (AVWP) was 320 msec and the minimal pacing cycle length maintaining 1:1 retrograde conduction (VAWP) was 400 msec. Single extrastimulus testing in the right atrium and the right ventricular apex leaded to a sustained narrow complex tachycardia. The arrhythmia was a short PR- long RP tachycardia with following characteristics: cycle length=376 msec, AH=141 msec, HV=42 msec, VA=200 msec, HA (HRA) =236 msec, HA (His) =243 msec and eccentric atrial activation during the arrhythmia (Figure 1). The arrhythmia was easily reproducible with stable hemodynamic.

A ventricular extrastimulus delivered at RV septum in the inflow region synchronous with His activation during the arrhythmia neither advanced nor delayed the subsequent atrial activation. Earlier ventricular premature beat at the RV septum terminated the arrhythmia (the His was not refractory) without conduction to the atrium. Right ventricular apical pacing at a cycle length just shorter than the tachycardia cycle length showed the same retrograde atrial activation sequence as during the arrhythmia.

**Figure 2:**
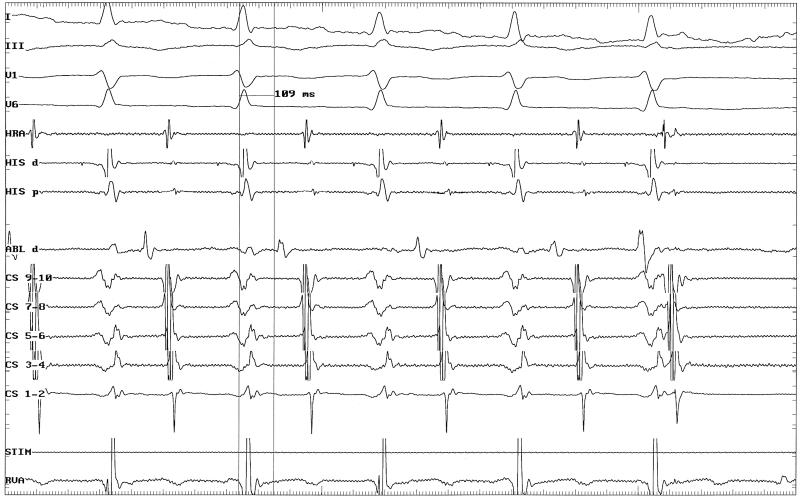
Surface leads and intracardiac electrograms during radiofrequency catheter ablation of the accessory pathway are shown. Application of energy led to termination of arrhythmia. The VA interval at successful ablation site is 109 msec. Recording speed: 100 mm/sec.

Subsequently, the right atrioventricular annulus was mapped in the tachycardia and the shortest VA interval was noted at the posterolateral of tricuspid annulus. The radiofrequency current was delivered to this site at the ventricular aspect of tricuspid annulus and resulted in termination of the arrhythmia within 5 seconds. The successful ablation site had a VA interval of 109 msec (Figure 2).

**Figure 3:**
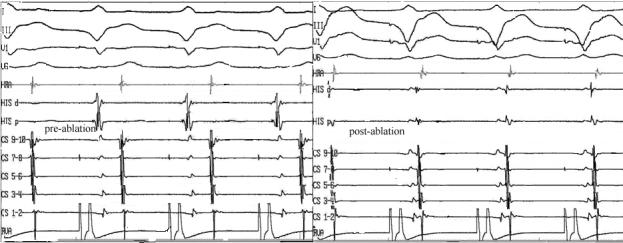
Surface leads and intracardiac electrograms during ventricular pacing (cycle length: 580 msec) before and after ablation of the accessory pathway are shown. Atrial and ventricular electrograms have been fused after ablation of the accessory pathway. Recording speed: 75 mm/sec.

Following this ablation, programmed atrial and ventricular stimulation failed to induce any arrhythmia and retrograde atrial activity sequence during burst ventricular pacing was concentric. Comparing retrograde atrial activity sequence before ablation with retrograde atrial activity sequence after ablation in the same pacing cycle length showed a significant decrease in VA interval after ablation of the accessory pathway (Figure 3). What is the mechanism?

## Discussion

The differential diagnosis of a short PR- long RP tachycardia should include atypical AVNRT, orthdromic AVRT involving a slowly conducting retrograde accessory pathway and atrial tachycardia.

There are a variety of pacing maneuvers which can be useful in differentiating these arrhythmias. One of the most important methods of diagnosing the presence and participation of retrogradely conducting bypass tracts during SVT is the ability of a ventricular extrastimulus to depolarize the atrium, with the same atrial activation sequence as supraventricular tachycardia when the His bundle is refractory. The site of stimulation relative to the site of the bypass tract and the rate of the tachycardia are the main determinants of the ability to preexcite the atrium.  Because the slowly conducting bypass tracts have decremental conduction property, the response to premature ventricular stimuli results in slowing of VA conduction which can retard the return cycle [1].

Burst pacing from the right ventricle at a cycle length just shorter than the tachycardia cycle length is another maneuver. If ventricular pacing produces the same retrograde activation sequence, a bypass tract can be diagnosed.  Also, measuring the delta H- A interval (H-A interval during tachycardia minus H-A interval during pacing) can distinguish tachycardia that is due to AVNRT from septal bypass tract [2].

Based on the electrophysiologic findings, the patient had a slowly conducting right posterolateral accessory pathway and the arrhythmia was an orthdromic AVRT. The concealed accessory pathway that conducts in retrograde fashion with a decremental property usually is located in posterior region near the posterior interatrial septum. In our case, the accessory pathway was located at posterolateral region of tricuspid annulus. Ticho et al reported four patients with the permanent form of junctional tachycardia in whom slowly conducting retrograde accessory pathways were located in sites other than the posteroseptal area. It was in the right free wall in two patients, in the right anterior septum in one patient and in the left free wall in one patient [3].

In our case, eccentric atrial activation both during the arrhythmia and during ventricular pacing was diagnostic of a concealed bypass tract.

The ventricular extrastimulus could not advance or delay the subsequent atrial activation. It might be due to the location or slow conduction property of the accessory pathway.

Before ablation of the accessory pathway, retrograde atrial activation sequence had been eccentric and conduction had been via the accessory pathway with slowly conducting property but after that, retrograde atrial activation sequence was concentric with short VA interval. Since the retrograde His signal could not be seen, HA interval during RV pacing could not be determined. According to post-ablation study, VA conduction was decremental and no arrhythmia was inducible so the presence of another accessory pathway is unlikely and it seems that retrograde atrial conduction was via fast pathway. But, what is the reason that retrograde atrial conduction had been predominantly via the accessory pathway before we ablated it?

We reviewed the intracardiac electrograms. During the procedure, we had to sedate the patient. Before sedation of the patient, retrograde atrial activation had been concentric with short VA interval but after that, retrograde atrial activation was eccentric. It could be related to the decrease of sympathetic activity and catecholamine release after sedation of the patient. Because sympathetic stimulation could enhance AV node conduction and shorten its effective refractory period, the impulses had been conducted to atrium via fast pathway. Although slowly conducting accessory pathways behave like AV node in many respects, they have quantitatively differing response to autonomic and pharmacologic manipulation. It is similar to different responses of slow and fast pathways to autonomic stimulation in patients with AVNRT. For example, adrenergic stimulation tends to shorten ERP of fast pathway (both antegrade and retrograde) to a greater extent that of the slow pathway [4].

The persistence of conduction via the accessory pathway can be explained with repetitive concealed antegrade penetration of AV node by the impulse reaching the atrium, after being conducted retrogradely via the accessory pathway.

In conclusion, post-ablation retrograde atrial activation sequence was concentric and conduction was via AV node (fast pathway) so after ablation of the accessory pathway, VA interval was shortened and it seems that atrial and ventricular electrograms were fused.

## List of Abbreviations

AH interval: atrial-His interval
AVNRT: atrioventricular nodal reentrant tachycardia
AVRT: atrioventricular reentrant tachycardia
AVWP: atrioventricular wenckebach point
HA interval: His-atrial interval
HRA: high right atrium
HV interval: His-ventricle interval
VA interval: ventriculoatrial interval
VAWP: ventriculoatrial wenckebach point
